# Caproate production from Enset fiber in one-pot two-step fermentation using anaerobic fungi (*Neocallimastix cameroonii* strain G341) and *Clostridium kluyveri* DSM 555

**DOI:** 10.1186/s12934-023-02224-w

**Published:** 2023-10-20

**Authors:** Nebyat Seid, Katrin Ochsenreither, Anke Neumann

**Affiliations:** 1 Electrobiotechnology, Institute of Process Engineering in Life Science 2, Karlsruhe Institute of Technology (KIT), 76131 Karlsruhe, Germany; 2 School of Chemical and Bio Engineering, Addis Ababa Institute of Technology, Addis Ababa University, P.O.B: 1176, Addis Ababa, Ethiopia; 3 Department of Chemical and Process Engineering, Karlsruhe Institute of Technology (KIT), 76131 Karlsruhe, Germany

**Keywords:** Enset fiber, One-pot two-step fermentation, Caproate, *N. cameroonii* strain G341, *C. Kluyveri*, Anaerobic fungi, Co-culture

## Abstract

**Background:**

Lignocellulosic biomass plays a crucial role in creating a circular bioeconomy and minimizing environmental impact. Enset biomass is a byproduct of traditional Ethiopian Enset food processing that is thrown away in huge quantities. This study aimed to produce caproate from Enset fiber using *Neocallimastix cameroonii* strain G341 and *Clostridium kluyveri* DSM 555 in one-pot two-step fermentation.

**Results:**

The process started by growing *N. cameroonii* on Enset fiber as a carbon source for 7 days. Subsequently, the fungal culture was inoculated with active *C. kluyveri* preculture and further incubated. The results showed that *N. cameroonii* grew on 0.25 g untreated Enset fiber as the sole carbon source and produced 1.16 mmol acetate, 0.51 mmol hydrogen, and 1.34 mmol formate. In addition, lactate, succinate, and ethanol were detected in small amounts, 0.17 mmol, 0.08 mmol, and 0.7 mmol, respectively. After inoculating with *C. kluyveri*, 0.3 mmol of caproate and 0.48 mmol of butyrate were produced, and hydrogen production also increased to 0.95 mmol compared to sole *N. cameroonii* fermentation. Moreover, after the culture was supplemented with 2.18 mmol of ethanol during *C. kluyveri* inoculation, caproate, and hydrogen production was further increased to 1.2 and 1.36 mmol, respectively, and the consumption of acetate also increased.

**Conclusion:**

A novel microbial cell factory was developed to convert untreated lignocellulosic Enset fiber into the medium chain carboxylic acid caproate and H_2_ by a co-culture of the anaerobic fungi *N. cameroonii* and *C. kluyveri*. This opens a new value chain for Enset farmers, as the process requires only locally available raw materials and low-price fermenters. As the caproate production was mainly limited by the available ethanol, the addition of locally produced ethanol-containing fermentation broth (“beer”) would further increase the titer.

**Graphical Abstract:**

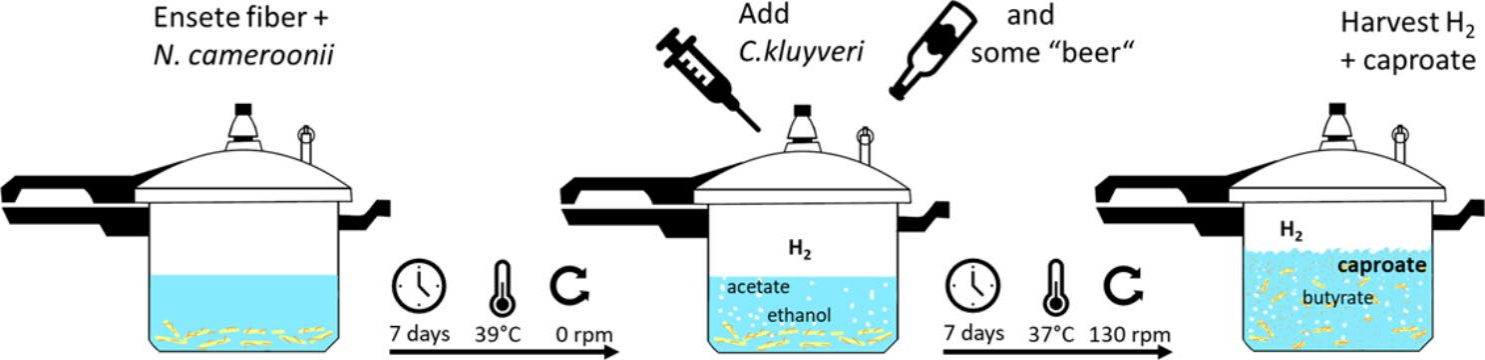

**Supplementary Information:**

The online version contains supplementary material available at 10.1186/s12934-023-02224-w.

## Background

International Energy Agency (IEA) bioenergy task 42 defines a biorefinery concept as a process that converts biomass into a variety of valuable products such as chemicals, materials, energy, food, and feed. The goal is to expand the range of products made from biomass to create other valuable products [1, 2]. The use of renewable resources is crucial for sustainable development and minimizing environmental impact [3]. Lignocellulosic biomass is the most attractive resource due to its plentiful availability, low cost, and lack of competition with food [4]. An enormous amount of byproduct known as Enset biomass is thrown away during traditional Ethiopian Enset food processing. It is estimated that more than twenty million Ethiopians are dependent on the Enset plant for their food supply [5, 6]. A major source of cellulose from the Enset plant is the Enset fiber, midrib, and leaf sheath peel, which contain 67.1%, 40%, and 34.1% cellulose, respectively [7]. Enset fiber can be used in a number of traditional products such as ropes, sacks, mats and bags [8]; however, the possibility of turning this waste into high value industrial products would strengthen the bioeconomy. Recent studies have shown that Enset biomass is a very promising lignocellulosic biomass for biobutanol [7] and pulp [9] production. However, to produce biofuel from Enset biomass, the material must undergo a degradation process that includes chemical and physical pretreatments before being hydrolyzed by enzymes. As a result, there is a substantial loss of biomass, environmentally unfriendly chemicals are used, and the process is energy intensive and expensive [10].

Research showed that anaerobic fungi can utilize lignocellulosic biomass without any pretreatment process and produce valuable products. As they grow, anaerobic fungi penetrate lignocellulosic biomass with their hyphal tips and degrade it with the enzyme complexes they produce [11, 12]. Anaerobic fungi can be extracted from the alimentary tract, saliva, and feces of a variety of herbivorous animals [13]. From the feces of zoo animals, Stabel M. et al. [14] isolated six different anaerobic fungal strains, including *Neocallimastix cameroonii, Pecoramyces ruminantium, Caecomyces spec., Khoyollomyces ramosus, Orpinomyces joyonii*, and *Aestipascuomyces dubliciliberans*. According to the study, of all isolates *Neocallimastix cameroonii* strain G341 proved to be the most efficient organism for utilizing wheat straw and was capable of producing acetate, succinate, ethanol, hydrogen, formate and lactate [15]. These products by itself can be already used to produce other bulk industrial chemicals [16], however, industrializing the use of mixed fermentation products may be challenging due to expensive and energy-intensive separation methods [17, 18]. Moreover, the market value of these products is not high compared to other products available on the market. To make this process economically more attractive, the products can be transformed into higher value products through the chain elongation process.

In chain elongation process, volatile fatty acids are changed to more valuable medium chain fatty acids through anaerobic fermentation [18]. A medium chain fatty acid like caproate, with its low solubility and high energy content, is used for various applications, such as antibiotics, feed additives, and as a component of products such as fragrances, lubricants, paint additives, and pharmaceuticals [19, 20]. Researchers have conducted several studies on the production of caproate from mixed organic waste [19], food waste [18] and wastewater [21] using an open culture. However, due to an undefined consortium of microorganisms potentially involved in the process, it is possible that substrates might be degraded and unwanted products might be produced in a hardly controllable way [22]. In pure culture fermentation, *Clostridium kluyveri* is capable of utilizing acetate, ethanol, succinate, and hydrogen to produce caproate and other medium chain fatty acids [23, 24]. Therefore, fermentation metabolites of *N. cameroonii* strain G341 from lignocellulosic biomass could be a suitable substrate for *C. kluyveri* fermentation. The objectives of this study were to evaluate the growth of *N. cameroonii* on Enset fiber, and to develop and characterize a one-pot two-step fermentation process for caproate production using *N. cameroonii* strain G341 and *C. kluyveri* DSM 555 from Enset fiber. To date, no research has been conducted on the growth of *N. cameroonii* on Enset fiber as well as the production of caproate from Enset fiber using *N. cameroonii* and *C. kluyveri* in a single pot two-step fermentation.

## Results

### Determination of ***N. cameroonii*** growth on Enset fiber

**Fig. 1:**
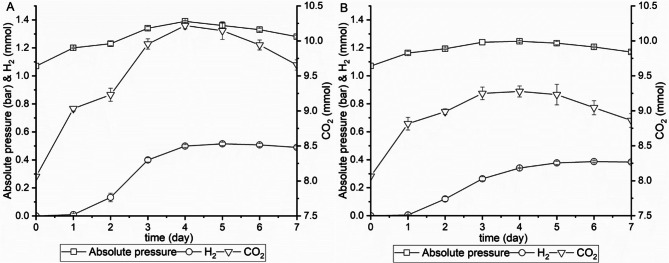
Gas production and pressure generated during *N. cameroonii* growth on 0.25 g (**A**) Enset fiber or (**B**) wheat straw. All values are means from triplicate bottles. Note the offset of the right (CO_2_) axis

To evaluate the growth of *N. cameroonii* on Enset fiber, and to analyze the fermentation products, the fungi were grown initially on 0.25 g Enset fiber as sole carbon source. Before fermentation, the bottles were filled with 8.06 mmol CO_2_ gas at atmospheric pressure (≈1.00 bar). As shown earlier by Theodorou M. et al. [25], pressure increase can be used as an indicator of growth and metabolite production, therefore, the bottle pressure was measured regularly. Figure 1 A shows the amount of gas produced by the anaerobic fermentation of *N. cameroonii* along with the absolute pressure built up. Within 24 h, an absolute pressure of 1.2 bar was developed and 0.97 mmol of CO_2_ were produced. As fermentation progressed, the gas composition in the bottle headspace changed to hydrogen and CO_2_, and a maximum pressure of 1.39 bar was generated on the fourth day. After 5 days, 0.51 mmol of hydrogen was produced with no significant changes in hydrogen quantity in the following days. However, there was a slight decrease in the amount of CO_2_ gas as well as the pressure. In the control experiments with 0.25 g wheat straw as substrate, the gas profiles were similar and the fungi developed a maximum pressure of 1.25 bar and 0.39 mmol hydrogen, which was lower than with Enset fiber (Fig. 1B).

**Fig. 2:**
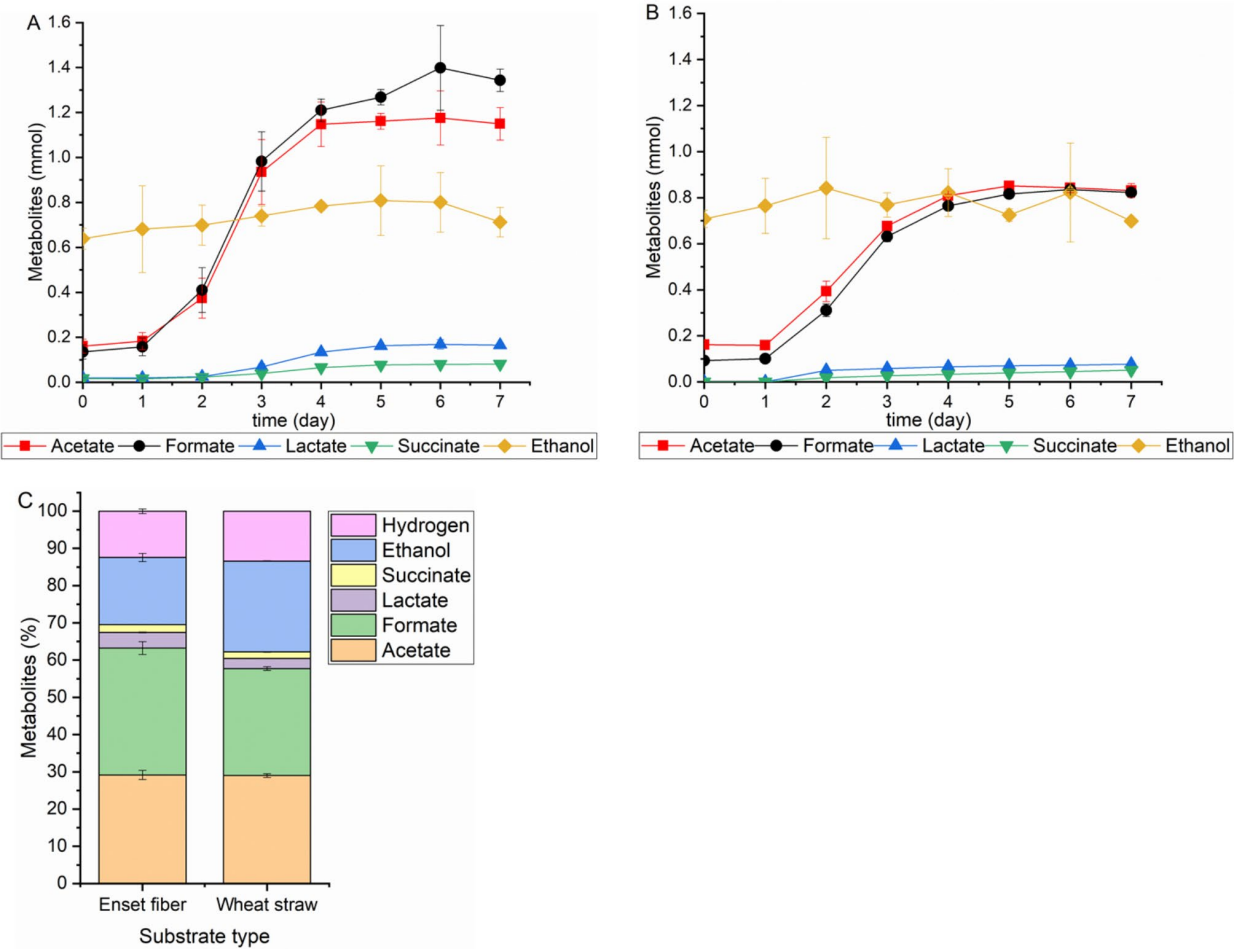
Soluble products of *N. cameroonii* growth on 0.25 g (**A**) Enset fiber, (**B**) wheat straw (**C**) mol percentage of all metabolites. All values are means from triplicate bottles

The amount of soluble products released during growth of *N. cameroonii* on 0.25 g of Enset fiber is shown in Fig. 2A. The cultures produced 0.37 mmol acetate and 0.41 mmol formate from Enset fiber within two days, however, lactate and succinate were not detected. After 5 days, the production was increased to 1.16 mmol acetate and 1.26 mmol formate. In addition, lactate and succinate were detected in small amounts, 0.16 mmol and 0.08 mmol, respectively. During the media preparation, the hemin solution containing ethanol was added to the media; but after 5 days of fermentation, the amount of ethanol increased to 0.8 mmol, indicating that ethanol was produced during the process. At 7 days, all amounts of metabolites remained constant except for formate, which increased to 1.34 mmol. For both substrates, the maximum total metabolite production rate was observed on the third day, but with Enset fiber a higher production rate than with wheat straw was calculated, 0.73 mmol/day compared to 0.49 mmol/day for wheat straw. At the end of the fermentation process, it was observed that the anaerobic fungi produced a total of 3.94 mmol of metabolites from Enset fiber, which was considerably higher than the 2.86 mmol of metabolites produced from wheat straw (Fig. 2A&B). When comparing the percentage of each metabolite, acetate, hydrogen, and succinate were all produced from Enset fiber in the same proportions as from wheat straw, 29, 13, and 1.9% (mmol/mmol), respectively. However, there was a significant difference in the percentage of formate (34% (mmol/mmol)) as well as lactate (4% (mmol/mmol)), and it was higher with Enset fiber than with wheat straw (Fig. 2C).

**Fig. 3:**
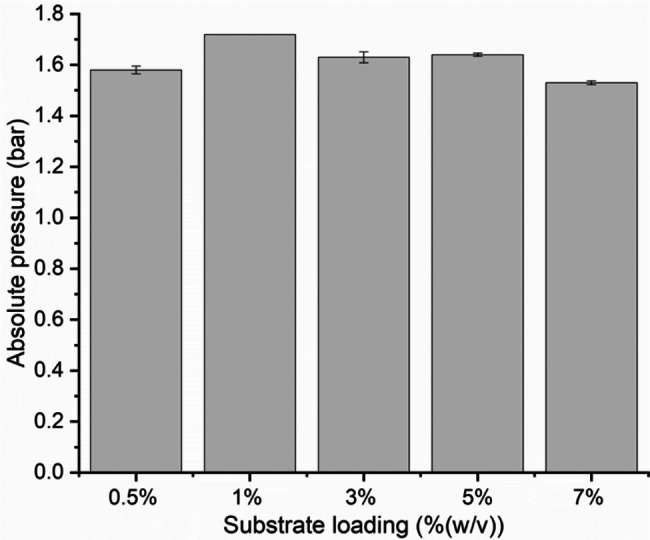
Pressure value measured at different substrate loading with Enset fiber during *N. cameroonii* growth at the end of fermentation. All values are means from triplicate bottles

**Fig. 4:**
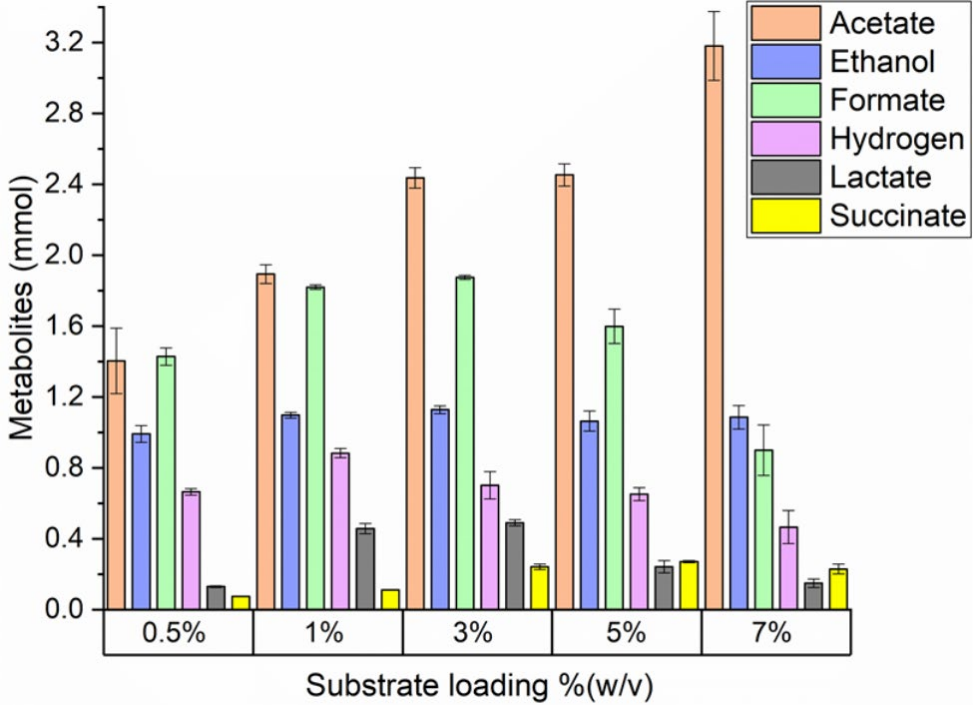
Fermentation products of *N. cameroonii* growth on different substrate loading of Enset fiber at the end of fermentation. All values are means from triplicate bottles

In addition, to investigate the influence of substrate loading on the growth of *N. cameroonii*, 0.5, 1, 3, 5 and 7% (w/v) of Enset fiber were tested. Figure 3 shows the pressure generated at the end of fermentation during *N. cameroonii* growth at different substrate loading of Enset fiber. The highest pressure of 1.72 bar was recorded with 1% (w/v) of Enset fiber, but the pressure decreased with increasing substrate loading. Statistical analysis with p < 0.05 showed a significant difference between different substrate loadings of Enset fiber on pressure development by *N. cameroonii* (see Additional file 1: Table S1). Moreover, the metabolite results shown in Fig. 4 indicate that all fermentation products increased when substrate loading was increased from 0.5 to 3% (w/v) except hydrogen. At 3% (w/v) substrate loading, acetate and formate increased to 2.44 mmol and 1.88 mmol. There was also an increase in lactate and succinate to 0.49 mmol and 0.24 mmol, respectively, but no significant difference in ethanol production was observed. On the other hand, a maximum hydrogen production of 0.88 mmol was observed at the substrate loading of 1% (w/v). All metabolites decreased at both 5 and 7% (w/v) substrate loadings, except acetate which increased to 3.18 mmol. The percentage of each metabolite produced during *N. cameroonii* growth showed that there was a similarity between the acetate and formate percentages of 30% (mmol/mmol) when the substrate loading was low, however, the acetate percentages increased to 53% (mmol/mmol) when substrate loading was high. Additionally, as substrate loading increased, the hydrogen percentage tended to decrease from 14 to 8% (mmol/mmol) as well (see Additional File 1: Figure S1).

### Caproate production from Enset fiber in one-pot two-step fermentation using ***N. cameroonii*** and ***C. kluyveri***

**Fig. 5:**
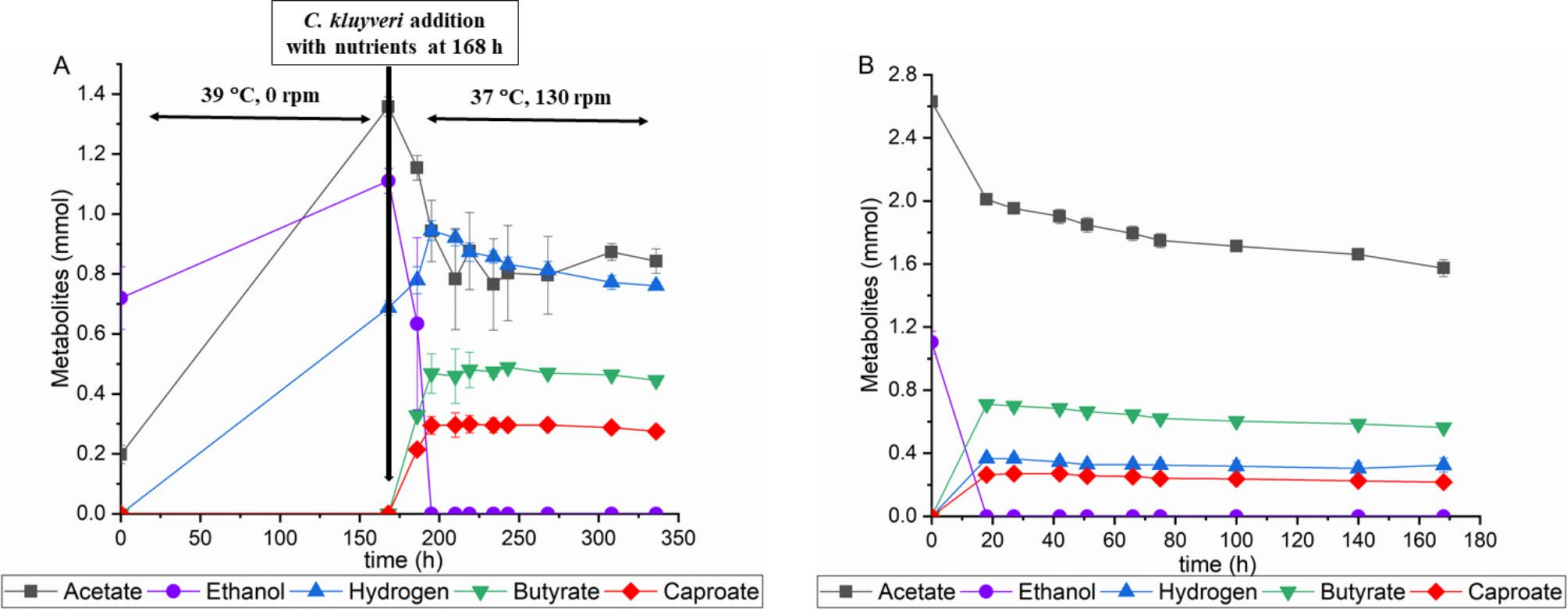
Fermentation products (**A**) one-pot two-step fermentation using Enset fiber without additional carbon source (**B**) *C. kluyveri* fermentation using acetate and ethanol as a carbon source. All values are means from triplicate bottles

The purpose of this study was to produce caproate directly from Enset fiber without any pretreatment or addition of external enzymes. *N. cameroonii* was cultivated on Enset fiber and then fermented with *C. kluyveri* to produce caproate in an one-pot two-step fermentation. Figure 5 A shows the metabolites produced in one-pot two-step fermentation using *N. cameroonii* and *C. kluyveri* from Enset fiber as sole carbon source. In the first step fermentation, *N. cameroonii* grew on 0.25 g Enset fiber for 7 days and produced 1.36 mmol acetate, 0.69 mmol hydrogen, and 1.11 mmol of ethanol. Within 18 h of starting the second fermentation, *C. kluyveri* produced 0.21 mmol caproate and 0.33 mmol butyrate. After 42 h, a maximum of 0.3 mmol caproate and 0.46 mmol butyrate were produced and thereafter there was no significant difference in production. In addition, the hydrogen production increased to 0.95 mmol and ethanol was completely consumed within 27 h. However, the remaining 0.76 mmol of acetate and other metabolites from anaerobic fungi were not consumed at the end of fermentation. As a control experiment, 2.63 mmol acetate and 1.1 mmol ethanol were used as a substrate for the *C. kluyveri* fermentation, and after 42 h 0.27 mmol caproate, 0.68 mmol butyrate and 0.34 mmol hydrogen were produced (Fig. 5B). Similarly to the two-step fermentation, all ethanol was consumed at the end of the fermentation, but 1.57 mmol of acetate remained unconsumed.

**Fig. 6:**
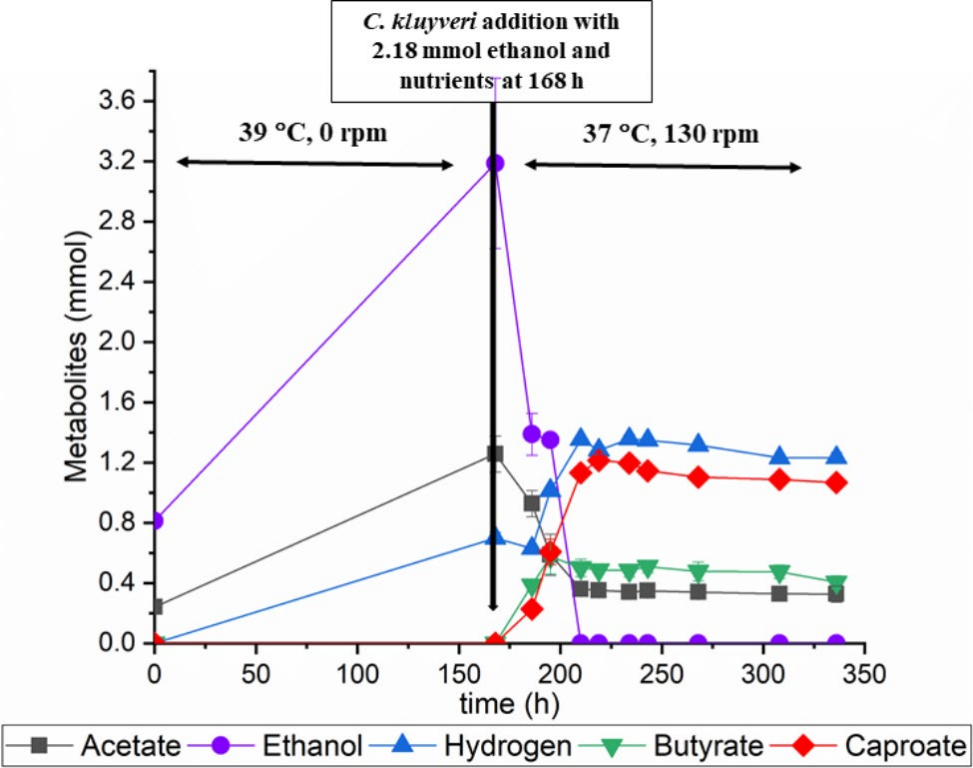
Metabolites from one-pot two-step fermentation using Enset fiber and with the addition of 2.18 mmol ethanol

In addition, separate experiments were conducted in which ethanol was added during *C. kluyveri* inoculation in the two-step fermentation to maximize acetate consumption and study the effect of ethanol. Figure 6 shows the results of two-step fermentation using Enset fiber and with the addition of 2.18 mmol ethanol. After second fermentation, there was no difference in the production of metabolites during the first 27 h of the experiment compared to previous experiments. However, the fermentation products after 42 h were significantly different from the previous experiment, more caproate was found than butyrate, 1.13 mmol and 0.5 mmol, respectively, and hydrogen production increased to 1.35 mmol. It was also found that all ethanol was consumed within 42 h and only 0.32 mmol acetate was not consumed.

### Carbon balance

Carbon balance estimation was performed for *N. cameroonii* growth on Enset fiber, and for one-pot two-step fermentation using *N. cameroonii* and *C. kluyveri* without additional carbon source (Table 1). In a study using Enset fiber as carbon source for growing *N. cameroonii*, 86.3% of the carbon was recovered as metabolites. The remaining carbon in the Enset fiber might not be degraded by anaerobic fungi. In the two-step fermentation, *C. kluyveri* achieved 94.1% carbon recovered as metabolites after the fungi were grown on Enset fiber, therefore it is likely that the missing carbon is bacterial cell biomass.

**Table 1:** Carbon balance for *N. cameroonii* growth on Enset fiber, and two-step fermentation using *N. cameroonii* and *C. kluyveri* on Enset fiber

Compound	Carbon per compound (mmol) for *N. cameroonii* growth on Enset fiber		Carbon per compound (mmol) for two-step fermentation ^a^	
	Substrates	Products	Substrates	Products
Enset fiber	8.58			
Acetate		2.22	2.72	1.69
Ethanol	1.28^b^	1.43	2.22	0
Succinate		0.32	0.44	0.24
Lactate		0.49	0.27	0.25
Formate		1.34	1.14	1.11
CO_2_	8.06	9.66	9.19	8.32
Caproate				1.65
Butyrate				1.78
Sum of carbon	17.92	15.47	15.97	15.03
Carbon recovery (%)^c^	86.3		94.1	

^a^ The two step fermentation was without addition of ethanol; ^b^ The amount of ethanol came from the hemin solution in the medium; ^c^ Carbon recovery (%)= (Sum of carbon in products/ Sum of carbon in substrate)×100%

## Discussion

### Anaerobic fungal growth on Enset fiber

Anaerobic fungi release carbohydrate-active enzymes (CAZymes) such as cellulases and hemicellulases that can degrade lignocellulosic biomass, and produce several organic acids, and hydrogen as byproducts of its growth process [26]. In this study, we observed that *N. cameroonii* grew on Enset fiber and produced acetate, succinate, ethanol, hydrogen, formate and lactate. During the fermentation process, the pressure in the headspace of the culture bottles increased due to the build-up of fermentation gases within the headspace of the bottles and indicated that anaerobic fungal growth was taking place [25]. Growth and production of metabolites by *N. cameroonii* started within 24 h on Enset fiber and remained constant after 5 days, suggesting that the growth process was completed by this time. Previous studies showed that after inoculation, the fungi were able to colonize and grow on the biomass by developing rhizoids, which then became a highly branched rhizoid system within 24 h [26, 27]. Stabel M. et al. [14] characterized the growth of *N. cameroonii* on different substrates such as wheat straw, cellobiose, xylan, cellulose, starch, pectin, chitin, inulin, alginate, maltose, sucrose, lactose, glucuronic acid, and monosaccharides. They found that *N. cameroonii* was capable of growing on all substrates except arabinose, chitin, alginate, galactose, ribose, and glucuronic acid, and produced hydrogen, acetate, succinate, ethanol, formate and lactate, when grown on these substrates. The study concluded that on wheat straw more hydrogen (0.35 mmol) was produced compared to monosaccharide substrates, which was less than when Enset fibers were used as the substrate. Furthermore, in our study, the amount of CO_2_ increased until the fourth day, similar to what Borneman W. et al. [28] observed in their study of *Neocallimastix sp*. Strain RI grown on Italian ryegrass hay. However, it was not sure whether it came from bicarbonate buffer or anaerobic fungal growth [25]. It is necessary to conduct further research to confirm the cause of the CO_2_ sources.

One of the factors that affect the metabolite production and the growth of *N. cameroonii* is the type of substrate. *N. cameroonii* grew better on Enset fiber and produced more metabolites than with wheat straw, compared to the control experiment shown in Fig. 2A&B. This could be due to the structural difference in the lignocellulosic biomass. It has been found that Enset fiber contains 67.1% cellulose, 15.6% hemicellulose, and 5.1% lignin while wheat straw contains 35–45% cellulose, 20–30% hemicellulose, and around 15.5% lignin [7, 29]. A significant difference between Enset fiber and wheat straw can be seen in the lower percentage of lignin and the higher percentage of cellulose, which could make Enset fiber a more suitable substrate for the fungi growth. Geoffrey L. et al. [30] conducted experiments in order to determine whether lignin is degraded by ruminal fungi, *Neocallimastix sp.* (strain LM-1), *Sphaeromonas sp.* (strain NM-1) and *Piromonas sp.* (strain SM-1), and the results showed that the lignin component of straw was not degraded. However, other researchers observed a high percentage of dry weight and lignin loss in perennial ryegrass stem cell walls [31], Bermudagrass leaf, cordgrass fiber [32], wood [33], and Italian ryegrass hay [34], when treated with different species of anaerobic fungi. However, most of the losses occurred due to the solubilization of lignin-polysaccharide complexes such as phenolic compounds [35]. In addition, there is some evidence that phenolic compounds might inhibit the growth of anaerobic rumen fungi [33]. However, further investigations should be carried out on the cell wall degradation of lignocellulosic biomass and on the effects of phenolic compounds on *N. cameroonii* growth.

There is no doubt that anaerobic fungal growth is highly dependent on the proper substrate loading, and an excessive solid substrate in the system can reduce the anaerobic fungi growth. In the current study, *N. cameroonii* growth on Enset fiber was observed at higher substrate loading. Even though there was a mixing problem due to the high amount of solid content in the bottles, at 7% (w/v) substrate loading of the Enset fiber a pressure of 1.53 bar was developed. However, the carbon balance calculation showed that at maximum substrate loading, the fungi’s carbon conversion efficiency was minimized from 98.8 to 16.5% (Table 2). 3% (w/v) was a suitable substrate loading to obtain a higher amount of soluble products, whereas 1% (w/v) substrate loading was more suitable for producing maximum hydrogen. In addition, it was observed that the maximum pressure exerted by *N cameroonii* on Enset fiber varied between 1.53 and 1.72 bar (Fig. 3). Thereafter, metabolism shifted from gaseous to soluble products, possibly due to the thermodynamic limitations of the microorganism [15]. The results of this study were similar to those of Zhu et al., [36], who conducted batch fermentation ranging from 5 to 80 g dry matter/L of wheat straw to determine the growth of *Neocallimastix hurleyensis*, indicating that about 45% wheat straw were degraded by the fungi at 5 g dry matter/L of substrate loading, whereas degradation efficiency was lowered with 10–80 g dry matter/L. There could be a number of reasons for this, including mass transfer between nutrients and fungi being limited [37], which might affect the rate at which nutrients are transferred to fungi. In the case of higher substrate loading levels, we observed that homogeneity of the substrate was causing a problem and the fungus only grew on one side of the bottle. In addition, according to the study [15], the mass transfer problem in *N. cameroonii* fermentation could also occur between the liquid and the gas phases, where the produced hydrogen gas might accumulate on the fungus mat and thereby inhibit its growth. To consolidate the reason behind the conclusions, further research should be conducted on product inhibition of *N. cameroonii* growth.

**Table 2:** Carbon conversion efficiency for *N. cameroonii* growth on different substrate loading of Enset fiber

Compound	Carbon per compound (mmol)				
	0.5% (w/v)	1% (w/v)	3% (w/v)	5% (w/v)	7% (w/v)
Substrates					
Enset fiber	8.58	17.2	51.5	85.8	120.1
Ethanol^*^	1.28	1.28	1.28	1.28	1.28
CO_2_	8.06	8.06	8.06	8.06	8.06
Products					
Acetate	2.81	3.79	4.88	4.91	6.36
Ethanol	1.98	2.20	2.26	2.13	2.17
Succinate	0.30	0.45	0.97	1.09	0.92
Lactate	0.39	1.37	1.47	0.73	0.45
Formate	1.43	1.82	1.88	1.60	0.90
CO_2_	10.8	11.1	10.9	11.2	10.6
**Carbon recovery (%)**	98.8	78.3	36.7	22.7	16.5

*The amount of ethanol came from the hemin solution in the medium

### One-pot two-step fermentation

In this study, a novel approach was developed to produce caproate from Enset fiber in a two-step fermentation process using *N. cameroonii* and *C. kluyveri* in a single bottle. Using this method, Enset fiber was directly converted into caproate without pretreatment or the addition of an external enzyme. Using Enset fiber as a raw material for caproate production contributes to a circular bioeconomy that creates sustainable systems. Furthermore, the two-step fermentation process could have several advantages for industrial applications, including reducing equipment costs because it used a single reactor, as well as chemical costs, as fewer reducing agents, antibiotics, and pH-adjusting chemicals are needed. Several studies have shown that caproate can be produced in one bioreactor by merging the chain elongation process with syngas fermentation [38, 39]. However, one of the biggest challenges was the pH unsuitability of acetogens and chain-elongating microorganisms [40]. Researchers found that *C. kluyveri* growth was inhibited at an acidic pH, which is the ideal pH for acetogens strains to maximize solvent production [41, 42]. During this study, a neutral pH was used in a one-pot two-step fermentation process that was suitable for the growth of both *N. cameroonii* and *C. kluyveri*, making it ideal for caproate production.

In this study, anaerobic fungi were able to directly convert Enset fiber into acetate, succinate, ethanol, hydrogen, formate, and lactate. The organic compounds collected from anaerobic fungal fermentation were successfully converted into high-value medium-chain carboxylates (MCC) such as caproate and butyrate. In addition, it was observed that hydrogen production was maximized in a two-step fermentation process compared to a single fermentation of anaerobic fungi (Fig. 5A). In this study, *C. kluyveri* metabolites production stopped around 42 h and entered a stationary phase, showing a similar growth process as in the study by Yin Y. et al. [20]. Furthermore, there was faster consumption of acetate and ethanol, as well as a small amount of succinate consumption. *C. kluyveri* is known to be able to consume a wide range of substrates and studies on this strain have demonstrated that succinate can also be consumed [43]. In addition, organic compounds such as formate and lactate, produced by anaerobic fungi, accumulated in the bottles. It appears that none of the metabolites accumulated from anaerobic fungal fermentation inhibited *C. kluyveri* growth.

Numerous studies have been conducted to evaluate the potential of anaerobic fungi for co-cultivation with other strains. Li Y. et al. [44] studied a co-culture experiment between an anaerobic fungus (*Pecoramyces* species) and a methanogen (*Methanobrevibacter* species), and the results showed that methane can be produced from various parts of corn stover through coculturing. However, in another study, Dollhofer V. et al. [45] showed that the co-cultivation of anaerobic fungi with mixed cultures from biogas plants faced many challenges, including unfavorable conditions in the digester due to extreme temperatures and long retention time. Moreover, it is also possible that there might be competition between the strains for nutrients and substrates [46]. In this study, besides suitable fermentation conditions such as an anaerobic environment, optimal pH, and temperature for the two strains, apparently, there was no competition between the anaerobic fungi and *C. kluyveri* for the availability of nutrients and substrates, since the strains were added sequentially and the nature of metabolic pathways between them were different. In the two-step fermentation process, *N. cameroonii* degraded lignocellulosic biomass by carbohydrate-active enzymes (CAZymes) to monosaccharides such as glucose and xylose, which are then metabolized through the Embden-Meyerhof pathway to generate energy carrier compounds. After that, pyruvate metabolism takes place in the cytosol and hydrogenosomes, producing various metabolic end products like acetate, succinate, ethanol, hydrogen, formate, and lactate [15, 47]. The end products of anaerobic fungi are important substrates in *C. kluyveri* metabolism and are involved in energy production and biosynthesis of cellular components [48]. *C. kluyveri* can break down fatty acids into medium-chain carboxylates (MCC) through the reverse β-oxidation pathway [49]. Despite this, it has been found that anaerobic fungi are unable to produce high concentrations of ethanol, which can be used as a source of energy, carbon, and reducing equivalents for the production of caproate through a reverse β-oxidation pathway [24].

Some studies have shown that hydrogen can potentially act as an electron donor in the production of caproate from acetate [20, 50]. However, the strain in this study did not use hydrogen or lactate as electron donors, likewise, previous results have shown that ethanol cannot be substituted by neither hydrogen nor lactate as electron donors in pure cultures of *C. kluyveri* [23, 24]. Ding, H. et al. [51] studied the metabolic pathways of caproate formation in *C. kluyveri* and concluded that caproate is not formed through hydrogenotrophic processes but by hydrogenogenic processes. Moreover, we observed that metabolite production ceased after ethanol was consumed completely, and 0.76 mmol of acetate produced by anaerobic fungi remained unconsumed (Fig. 5A). Similar results were also observed in the control experiment, where 2.63 mmol acetate and 1.1 mmol ethanol were used as a substrate for *C. kluyveri* fermentation, however, even though 1.57 mmol of acetate was present in the media, the strain could only produce 0.27 mmol of caproate (Fig. 5B). This shows that as soon as the ethanol content was reduced to zero, the strain stopped producing caproate since a biochemical reaction is thermodynamically only possible when an electron donor is available [52]. In addition, a comparative study of two-step fermentation with and without the addition of ethanol showed that the caproate yield increased significantly, reaching 0.27 mmol/mmol when ethanol was added to the fermentation, with a maximum productivity of 1.31 mmol/day including butyrate and hydrogen. In contrast, without ethanol, the caproate yield and maximum productivity were 0.18 mmol/mmol and 0.59 mmol/day, respectively. However, in the case of butyrate yield, the yield reduced from 0.26 mmol/mmol to 0.12 mmol/mmol when ethanol was added. This proved that ethanol is necessary to maximize the caproate yield as well as for the metabolic pathway to shift toward an increased amount of caproate. According to Grootscholten et al. [53], the addition of ethanol accelerates the chain elongation process in multiculture acidification reactors from municipal solid wastes of organic fraction. Furthermore, a higher ethanol/acetate ratio turned out to be more favorable for caproate production, while butyrate production was more favorable at lower ethanol/acetate ratios [24]. In addition, the hydrogen gas produced during two-step fermentation holds promise as a valuable substrate for syngas fermentation, which consists of hydrogen, carbon dioxide, and carbon monoxide, with the aim of producing ethanol and feeding it back into the two-step fermentation process as needed for caproate production. This strategic use has the potential to improve the sustainability of the two-step fermentation process, enabling it to fulfill ethanol demand. Research has confirmed the ability of *Clostridium ljungdahlii* to perform syngas fermentation, leading to the production of ethanol [54]. Moreover, a deeper investigation should be conducted into optimizing the fermentation process of anaerobic fungi to maximize the amount of acetate and ethanol they produce from Enset fiber.

## Conclusions

In this study caproate production from Enset fiber was realized in one-pot two-step fermentation using *N. cameroonii* and *C. kluyveri.* The results showed that *N. cameroonii* grew better on Enset fiber and produced more metabolites than with wheat straw. A successful method was developed to convert anaerobic fungi metabolites into caproate and butyrate. In addition, with this method, hydrogen production increased, and *C. kluyveri* growth was not inhibited by either *N. cameroonii* growth or accumulated organic compounds. Utilizing inexpensive raw materials for caproate production, such as Enset fiber, is an effective way to create a sustainable system and minimize environmental impacts. This novel approach provides valuable data for future research and industrial applications. However, further studies need to be done to maximize the yield and productivity of caproate production, such as optimizing fermentation parameters for one-pot two-step fermentation and conducting feasibility studies on the process to ensure that it can be applied to industrial processes.

## Materials and methods

### Enset fiber preparation

Enset fiber was collected from Wolkite, Ethiopia, and was prepared according to previous instructions [7]. It was dried for four days in the sun, cut into 6 cm pieces with scissors, and ground using a knife mill. The pulverized sample was sieved to 1 mm particle size and stored in a plastic bag.

### Anaerobic fungi cultivation and growth culture conditions

In a previous study, Stabel M. et al. [14] isolated *Neocallimastix cameroonii* strain G341 from giraffe feces. The strain was grown anaerobically in 250 mL serum bottles containing 50 mL of sterilized basal minimal medium and 0.25 g Enset fiber (5 g/L) as a carbon source corresponding to a substrate loading of 0.5% (w/v). 1 L minimal medium consisted of 150 mL of salt solution I (K_2_HPO_4_ (3.0 g/L)), 150 mL of salt solution II (KH_2_PO_4_ (3.0 g/L), (NH_4_)_2_SO_4_ (6.0 g/L), NaCl (6.0 g/L), MgSO_4_.7H_2_O (0.6 g/L), CaCl_2_.2H_2_O (0.6 g/L)), 2 mL of 0.05% (w/v) hemin solution (a mixture of 0.05 g hemin, 50 mL ethanol, and 50 mL of 0.05 M NaOH), 2 mL of 0.1% (w/v) resazurin solution, NaHCO_3_ (6 g/L), cysteine-HCl·H_2_O (1 g/L) and 10 mL of trace elements solution (a mixture of 0.25 g MnCl_2_.4H_2_O, 0.25 g NiCl_2_.6H_2_O, 0.25 g NaMoO_4_.2H_2_O, 0.25 g H_3_BO_3_, 0.20 g FeSO_4_.7H_2_O, 0.05 g CoCl_2_.6H_2_O, 0.05 g Na_2_SeO_3_.5H_2_O, 0.05 g NaVO_3_.4H_2_O, 0.025 g ZnSO_4_, 0.025 g CuSO_4_.2H_2_O, and 1 L 0.2 M HCl). The media was anaerobized by flashing with 100% CO_2_ until the color turned yellow, and the pH was corrected to 6.9 with 5 M NaOH. During anaerobization, a serum bottle containing 0.25 g of Enset fiber was filled with 49.5 mL of medium, enclosed with a rubber stopper and aluminum cap, and autoclaved. After autoclaving, 0.5 mL filter-sterilized vitamin solution (0.01 g/L thiamine, 0.2 g/L riboflavin, 0.6 g/L calcium pantothenate, 0.6 g/L nicotinic acid, 1.0 g/L nicotinamide, 0.05 g/L folic acid, 0.02 g/L vitamin B12, 0.2 g/L biotin, 0.1 g/L pyridoxamine, and 0.05 g/L p-aminobenzoic acid) was added [14, 55]. The main fermentation culture was inoculated with 10% (v/v) actively growing *N. cameroonii* preculture propagated every seven days for two consecutive cultivations before being used as a preculture. The bottle was incubated at 39 °C for 7 days in the dark in an incubator (Infors Thermotron, Infors AG, Bottmingen, Switzerland) without shaking. The pressure of the bottle was measured with manometer GMH 3100 Series (Greisinger, Mainz, Germany) at 39 °C, and 5 mL gas and 1.5 mL liquid samples were taken with needles every day and centrifuged at 10,000 rpm for 10 min [14]. 0.25 g wheat straw (5 g/L) was used as a substrate for the control experiment, and all experiments were performed in three replicates.

### Effect of substrate loading on ***N. cameroonii*** growth

The same conditions as mentioned above were used to cultivate *N. cameroonii* to investigate the effects of substrate loading. A total of 50 mL of media was prepared with Enset fiber at different substrate loadings (0.5, 1, 3, 5, 7% (w/v)) as carbon source, inoculated with 5 mL of anaerobic fungal culture, and incubated at 39 °C for 7 days [14]. At the beginning and the end of fermentation, 5 mL gas and 1.5 mL liquid samples were taken. All experiments were conducted in triplicate.

### Bacterial strain cultivation

Freeze-dried *Clostridium kluyveri* DSM 555 culture was acquired from the German Collection of Microorganisms and Cell Cultures, Braunschweig, Germany (DSMZ), and revived according to DSMZ protocol. The DSM-52 medium contained buffer stock solution (K_2_HPO_4_ (31 g/L), KH_2_PO_4_ (23 g/L), NH_4_Cl (25 g/L)), vitamin stock solution (B12 (0.1 g/L), p-aminobenzoic acid (0.08 g/L), biotin (0.02 g/L), nicotinic acid (0.2 g/L), D-Ca-pantothenate (0.1 g/L), pyridoxine-HCl (0.3 g/L), thiamine-HCl.2H_2_O (0.2 g/L)), yeast extract stock solution (100 g/L), Na_2_CO_3_ stock solution (100 g/L), Na_2_S.9H_2_O stock solution (100 g/L), L-Cysteine-HCl.H_2_O stock solution (100 g/L), and mineral stock solution (MgSO_4_.7 H_2_O (20 g/L), 100 mL of trace element solution, 100 mL of selenite-tungstate solution. The trace element solution SL-10 consisted of 10 mL of (25%) HCl in 1 L, FeCl_2_.4H_2_O (1.5 g/L), ZnCl_2_ (0.07 g/L), MnCl_2_.4H_2_O (0.1 g/L), H_3_BO_3_ (0.006 g/L), CoCl_2_.6H_2_O (0.19 g/L), CuCl_2_.2H_2_O (0.002 g/L), NiCl_2_.6H_2_O (0.024 g/L), Na_2_MoO_4_.2H_2_O (0.036 g/L). The selenite-tungstate solution was prepared by mixing NaOH (0.5 g/L), Na_2_SeO_3_.5H_2_O (0.003 g/L), and Na_2_WO_4_.2H_2_O (0.004 g/L). All the stock solutions were filter sterilized and anaerobized with 100% N_2_ gas. For the cultivation experiment, 10 g/L CH_3_CO_2_K and 1 g/L resazurin were added into 47.75 mL distilled water. The pH was corrected to 6.9 with 1 M NaOH/1 M HCl and filled into 250 mL serum bottles, enclosed with an aluminum cap and rubber stopper. Each bottle was flashed with a mixture of 80% N_2_ and 20% CO_2_ gases using needles and autoclaved. Then, after autoclaving, 1 mL of (99.5%) ethanol was injected with a needle and 1% (v/v) of each stock solution was added. 10% (v/v) of active culture with an OD of 0.8-1.0 was inoculated into bottles and incubated at 37 °C and 150 rpm. The culture was transferred three times before being used as a preculture [20].

### Two-step fermentation process

**Fig. 7:**
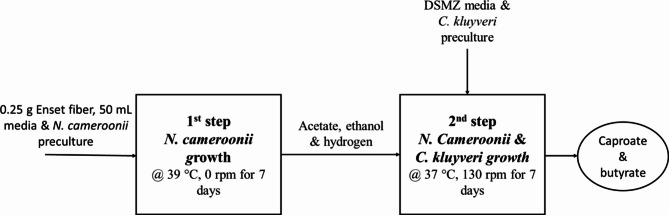
Caproate production from Enset fiber in two step fermentation using *N. cameroonii* and *C. Kluyveri*

*N. cameroonii* was first grown on 0.25 g Enset fiber at 39 °C for 7 days in 250 mL serum bottles containing 50 mL of the basal medium described above. Subsequently, the fungal culture was supplemented with 1% (v/v) sterilized DSM-52 stock medium without acetate, ethanol, and reducing agents. The bottle containing the grown fungal culture was then inoculated with 10% (v/v) of the actively growing *C. kluyveri* culture with an OD of 0.8-1.0 and incubated at 37 °C and 130 rpm for 7 days. During the second fermentation, the pressure was measured, and the 5 mL gas and 1.5 mL liquid samples were taken using needles (Fig. 7). In addition, to investigate the effect of ethanol in two-step fermentation, another experiment was conducted separately by supplementing the bottle with 2.18 mmol ethanol during *C. kluyveri* inoculation. As a control, *N. cameroonii* and *C. kluyveri* were grown separately using the standard media. All experiments were conducted in triplicate.

### Carbon balance calculations

The carbon balance calculation was estimated for *N. cameroonii* growth on Enset fiber, and one-pot two-step fermentation using *N. cameroonii* and *C. kluyveri* from Enset fiber without additional carbon source. Carbon recovery was calculated by balancing the carbon moles contained in all substrates and products (see Additional file 1: Table S2). The calculation was done without considering the amount of biomass from fungal and bacterial cells. In addition, the Enset fiber carbon content was estimated from its elemental composition, which was estimated by Seid N. et al. [7] with 41.2% (w/w) dry weight and calculated as follows:

1 $$\large \large \normalsize Amount\,of\,carbon\,in\,Enset\,fiber\,(mol)\,=\,\frac{mass\,of\,Enset\,fiber\,(g)\times 41.2 \%\,(\frac{g}{g})\,Carbon}{Molar\,mass\,of\,carbon\,(\frac{g}{mol})}$$

### Analytical methods

After inoculation, the gas pressure inside the bottle was monitored using a manometer with a needle. As anaerobic fungi grew and produced gases, the manometer recorded changes in pressure over time. By correlating gas pressure measurements with fungal growth, this method provided insights into anaerobic fungal growth [14]. Ultraspec 1100 pro spectrophotometer (Amersham Biosciences, Uppsala, Sweden) was used to measure the optical density (OD_600_) of bacterial cells. For liquid samples, the pH was measured by Profilab pH 597 (Xylem Analytics, Weilheim, Germany) and metabolites were determined by high-performance liquid chromatography (HPLC) in a 1100 Series System (Agilent Technologies, Waldbronn, Germany). HPLC was performed with a Rezex ROA Organic Acid H^+^ (8%) column at 55 °C, and 5 mM H_2_SO_4_ eluent with a flow rate of 0.6 mL/min [7]. The gas samples were analyzed by Micro GC Fusion® gas analyzer (Inficon, Bad Ragaz, Switzerland) with Porous Layer Open Tubular (PLOT) and Wall Coated Open Tubular (WCOT) capillary columns using Argon and Helium as carrier gases.

### Electronic supplementary material

Below is the link to the electronic supplementary material.

**Additional file 1: Table S1**. Output from ANOVA analysis in OriginPro 2021; the pressure developed by *N. cameroonii* on different substrate loading of Enset fiber. **Figure S1**. Percentage of all metabolites for *N. cameroonii* growth on different substrate loading of Enset fiber at the end of fermentation. **Table S2**. Carbon balance calculation and conversion factor for *N. cameroonii* growth on Enset fiber

## Data Availability

Not applicable.
