# Supplementary material for: Caproate production from Enset fiber in one-pot two-step fermentation using anaerobic fungi (Neocallimastix cameroonii strain G341) and Clostridium kluyveri DSM 555
Source: Microb Cell Fact. 2023 Oct 20;22:216. doi: 10.1186/s12934-023-02224-w (PMC10588050; doi:10.1186/s12934-023-02224-w)
Supplement: Supplementary file 1 — Additional file 1: Table S1. Output from ANOVA analysis in OriginPro 2021; the pressure developed by N. cameroonii on different substrate loading of Enset fiber. Figure S1. Percentage of all metabolites for N. cameroonii growth on different substrate loading of Enset fiber at the end of fermentation. Table S2. Carbon balance calculation and conversion factor for N. cameroonii growth on Enset fiber [file 12934_2023_2224_MOESM1_ESM.pdf]

Caproate production from Enset fiber in one-pot two-step fermentation using anaerobic fungi *(Neocallimastix cameroonii* strain G341*)* and *Clostridium kluyveri* DSM 555

Nebyat Seid [1, 2, *], Katrin Ochsenreither [3] and Anke Neumann [1, *]

[1]Electrobiotechnology, Institute of Process Engineering in Life Science 2, Karlsruhe Institute of Technology (KIT), 76131 Karlsruhe, Germany

[2] School of Chemical and Bio Engineering, Addis Ababa Institute of Technology, Addis Ababa University, P.O.B: 1176, Addis Ababa, Ethiopia

[3], Department of Chemical and Process Engineering, Karlsruhe Institute of Technology (KIT), 76131 Karlsruhe, Germany

[*]Correspondence: anke.neumann@kit.edu or nebyatabdu@gmail.com

Table S1 Output from ANOVA analysis in OriginPro 2021; the pressure developed by *N. cameroonii* on different substrate loading of Enset fiber

ANOVAOneWay (14/06/2023 08:04:22)

Descriptive Statistics

	N Analysis	N Missing	Mean	Standard Deviation	SE of Mean
0.5%	3	0	1.57667	0.01528	0.00882
1%	3	0	1.72	0	0
3%	2	1	1.63	0.04243	0.03
5%	3	0	1.64667	0.00577	0.00333
7%	3	0	1.53333	0.04933	0.02848

One Way ANOVA

Overall ANOVA

	DF	Sum of Squares	Mean Square	F Value	Prob>F
Model	4	0.06049	0.01512	18.90402	2.09592E-4
Error	9	0.0072	8E-4		
Total	13	0.06769			

Null Hypothesis: The means of all levels are equal.
Alternative Hypothesis: The means of one or more levels are different.
At the 0.05 level, the population means are significantly different.

Fit Statistics

	R-Square	Coeff Var	Root MSE	Data Mean
	0.89364	0.01745	0.02828	1.62071

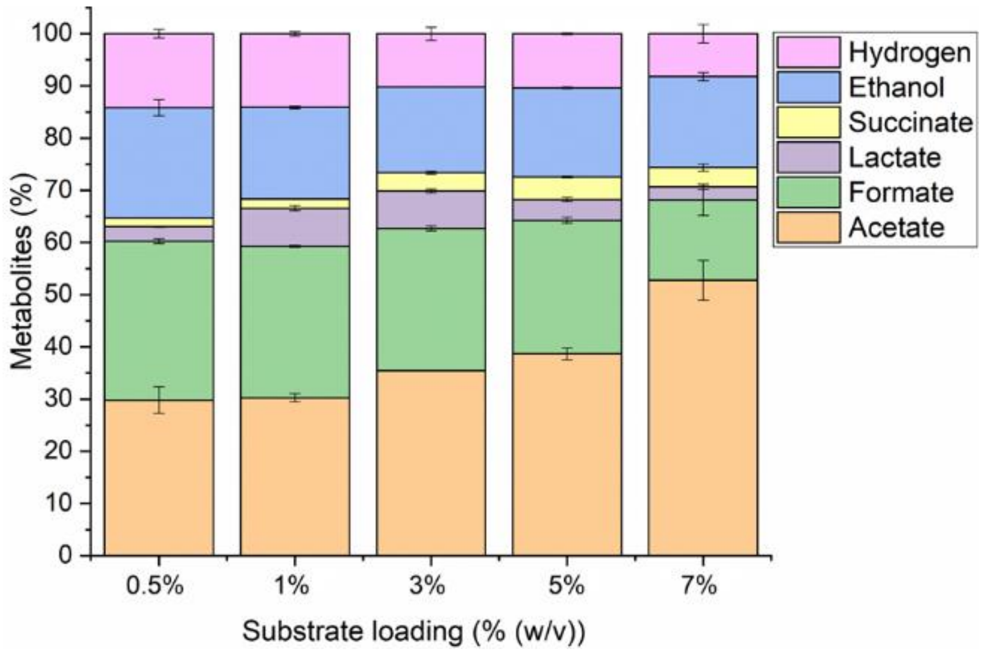

Figure S1 Percentage of all metabolites for *N. cameroonii* growth on different substrate loading of Enset fiber at the end of fermentation. All values are means from triplicate bottles

Table S2 Carbon balance calculation and conversion factor for *N. cameroonii* growth on Enset fiber

**Compound**	**Chemical formula**	**Molecular weight (g/mol)**	**mol Carbon/mol**	**Mole of Compound (mmol)**	**Carbon per compound (mmol)**
Initial substrates					
Enset fiber					8.58[a]
Ethanol	C_2H_6O	46.07	2	0.64	1.28[b]
CO_2	CO_2	44.01	1	8.06	8.06
Sum of carbon in substrate					17.92
Final products					
Acetate	$C_2H_4O_2$	60.05	2	1.11	2.22
Ethanol	C_2H_6O	46.07	2	0.71	1.43
Succinate	$C_4H_6O_4$	118.09	4	0.08	0.32
Lactate	$C_3H_6O_3$	90.08	3	0.17	0.49
Formate	CH_2O_2	46.03	1	1.34	1.34
CO_2	CO_2	44.01	1	9.66	9.66
Sum of carbon in product					15.47
Carbon recovery (%)[c]					86.33

[a] The mole of carbon in Enset fiber was calculated from 0.25g Enset fiber and carbon content of 41.2 %(w/w): [b] The amount of ethanol came from the hemin solution in the medium; [c] Carbon recovery (%) = (Sum of carbon in products/ Sum of carbon in substrate) × 100 %
